# Modulation of Ubiquitin Signaling in Innate Immune Response by Herpesviruses

**DOI:** 10.3390/ijms23010492

**Published:** 2022-01-01

**Authors:** Sandrine-M. Soh, Yeong-Jun Kim, Hong-Hee Kim, Hye-Ra Lee

**Affiliations:** 1 Department of Biotechnology and Bioinformatics, College of Science and Technology, Korea University, Sejong 30019, Korea; sandy391@korea.ac.kr (S.-M.S.); kyj1994@korea.ac.kr (Y.-J.K.); ghdtl02@korea.ac.kr (H.-H.K.); 2 Department of Laboratory Medicine, College of Medicine, Korea University, Seoul 136-701, Korea

**Keywords:** ubiquitin E3 ligases, deubiquitinases, herpesviruses, innate immunity, host-antiviral immune response

## Abstract

The ubiquitin proteasome system (UPS) is a protein degradation machinery that is crucial for cellular homeostasis in eukaryotes. Therefore, it is not surprising that the UPS coordinates almost all host cellular processes, including host–pathogen interactions. This protein degradation machinery acts predominantly by tagging substrate proteins designated for degradation with a ubiquitin molecule. These ubiquitin tags have been involved at various steps of the innate immune response. Hence, herpesviruses have evolved ways to antagonize the host defense mechanisms by targeting UPS components such as ubiquitin E3 ligases and deubiquitinases (DUBs) that establish a productive infection. This review delineates how herpesviruses usurp the critical roles of ubiquitin E3 ligases and DUBs in innate immune response to escape host-antiviral immune response, with particular focus on retinoic acid-inducible gene I (RIG-I)-like receptors (RLR), cyclic-GMP-AMP (cGAMP) synthase (cGAS), stimulator of interferon (IFN) genes (STING) pathways, and inflammasome signaling.

## 1. Introduction

The Herpesviridae are a family of large viruses that contain double-stranded DNA genomes and ubiquitously cause a range of human diseases of varying levels of severity [1]. They are classified into α-, β-, and γ-Herpesvirinae [2]. So far, nine human herpesviruses have been isolated, namely: herpes simplex virus 1 and 2 (HSV-1 and HSV-2) and varicella zoster virus (VZV) for α-Herpesvirinae, human cytomegalovirus (HCMV), human herpesvirus 6A (HHV6A), HHV6B, and HHV7 for β-Herpesvirinae, Epstein–Barr virus (EBV) and Kaposi’s sarcoma-associated herpesvirus (KSHV) for γ-Herpesvirinae [3]. Remarkably, the herpesviruses have two distinguished phases of life in order to sustain efficient lifelong persistency as well as their life cycles [4]. During the latent phase, herpesviruses only express a limited number of genes allowing them to effectively establish a lifelong persistency. Occasionally, under certain physiological conditions such as immunosuppression, herpesviruses can reactivate, then express all of their genes, leading to the production of progeny viruses [1]. Mounting data have shed more light on the underlying molecular mechanisms of the virus-host standoff in innate immune responses. This innate immune response is often initiated by pathogen recognition receptors (PRRs) that sense evolutionarily conserved molecular patterns of the pathogens, termed pathogen-associated molecular patterns (PAMPs) [5]. Until now, four types of PRRs have been reported; the Toll-like receptors (TLRs), nucleotide-binding oligomerization domain (NOD)-like receptors (NLRs), retinoic acid-inducible gene-I (RIG-I)-like receptors (RLRs), and cytosolic viral DNA sensors such as cyclic GMP-AMP synthase-stimulator of interferon genes (cGAS-STING) [6,7,8,9,10]. These different PRRs elicit intracellular signaling cascades that activate type I interferon (IFN) and pro-inflammatory cytokines, resulting in induction of innate immune response to eradicate the pathogen [11]. Due to their slow replication cycle and lifelong infection establishment, herpesviruses are therefore highly reliant on efficient immune evasion for survival. Thus, it is not surprising that herpesviruses have orchestrated various host proteins including ubiquitin proteasome system (UPS) which are involved in host surveillance, for their benefit. 

The UPS is completed through a cascade of three distinct enzymes that modify target proteins via conjugation of ubiquitin [12,13,14,15]. Ubiquitin (Ub), a highly conserved protein that harbors 76 amino acids, is activated by the E1 ubiquitin-activating enzyme which conducts adenylation of the ubiquitin C-terminal carboxyl group. Subsequently, the activated ubiquitin is transferred to the active site on the E2 ubiquitin-conjugating enzyme [16]. Finally, ubiquitin E3 ligase carries E2-conjugated ubiquitin to the target substrate by catalyzing the formation of a covalent isopeptide bond between substrate lysine and C-terminal of Ub molecule [17]. This protein modification can be either monoubiquitination or polyubiquitination by secondary ubiquitin linked to the different seven lysine linkages (K6, K11, K27, K29, K33, K48, and K63) or the N-terminus methionine (M1) [18]. Especially, only polyubiquitination on designated lysine residue, mainly on K29 and K48, caused proteasomal degradation, while other polyubiquitinations (on K6, K11, K27, K63, and M1) and monoubiquitination are involved in regulation of several processes like inflammation, innate immunity, endocytic trafficking, transcriptional regulation, and DNA repair [19,20,21]. The action of ubiquitin E3 ligases can be reversed by deubiquitinases (DUBs) which are enzymes that remove the ubiquitin molecule from its substrate, contributing to homeostasis [22]. Thus, ubiquitination and deubiquitination should be tightly controlled to fine-tune several cellular responses, including innate immune responses. In line with this point of view, herpesviruses have evolved ways to antagonize the host defense mechanisms by targeting ubiquitin E3 ligases and DUBs in order to minimize the host antiviral responses for establishment of productive infection [23]. This review will be spotlighting how herpesviruses subvert the critical roles of ubiquitin E3 ligases and DUBs in innate immune response to escape host-antiviral response. Especially, we would highlight the viral counteractions in the RLR, cGAS-STING pathways, and inflammasome signaling, but not in TLR pathway which is already highlighted in other papers.

## 2. E3 Ubiquitin Ligases and DUBs as Immune Modulators

### 2.1. E3 Ubiquitin Ligases (E3s)

Unlike E1 and E2 enzymes, the specific recognition of substrates mainly relies on E3 ubiquitin ligase. E3s, estimated to more than 600 different mammalian members, are currently classified into three major groups according to structural similarities of their domain and mechanisms of their catalytic activity. These groups are the really interesting new gene (RING)-type, the E3 ligase of the homologous to E6-associated protein (E6-AP) carboxyl terminus (HECT)-type, and the RING -between-RING (RBR)-type E3 ligase families [15,24,25,26,27,28]. The RING-type E3s are characterized by the presence of a zinc-binding domain known as RING or by a U-box catalytic domain, which accepts the same RING fold but does not enclose zinc. Depending on the number of RING domain, RING-type E3s can be classified into monomers, homodimers, or heterodimers [29]. In addition, some RING E3s are consisted of multi subunits, such as the cullin-RING ligases (CRLs) and the anaphase-promoting complex/cyclosome (APC/C). Specifically, CRLs comprise a greatly diverse class of ubiquitin ligases that are composed of a cullin scaffold, a RING domain catalytic subunit, different adaptors, and substrate receptors to assemble ubiquitin E3 machineries. The HECT-type E3s contain a HECT domain, which contains an active cysteine residue that forms a high-energy thioester bond with Ub before transferring it to the target substrate [26,28,30]. The substrate recognition of HECT-type E3s depends on protein–protein interactions at the N-terminal of their HECT domain [31]. Interestingly, the catalytic activity of HECTs is regulated by intramolecular interactions that maintain the protein in an auto-inhibited state, leading to response to numerous signals [32]. Based on their N-terminal extensions of the HECT domain, HECT type E3s can be further classified into three subfamilies: (1) Nedd4, which possess WW motifs, (2) HERC (HECT and RCC1-like domain) family, which contain one or more regulators of chromosome condensation 1 (RCC1)-like domains (RLDs), and (3) “other” HECTs that contain various domains [33]. The family of RBR-type E3s also catalyze Ub transfer via two steps: First, Ub is transferred to a catalytic cysteine on the E3. Subsequently, it transferred to the substrate. The RBRs encompass two predicted RING fingers, RING1 and RING2, and a central in-between-RINGs (IBR) zinc-binding domain. The RING1 domain binds ubiquitin-charged E2, subsequently transfers the Ub to the catalytic cysteine within RING2 [34,35,36]. Moreover, the RBR E3 ligases have other domains which have different individual structures to each member, ultimately contributing to their functions.

### 2.2. Deubiquitinating Enzymes (DUBs)

DUBs are proteases that catalyze either complete deubiquitination or trimming of Ub chains from proproteins, substrates, or their degradation remnants [37]. As mentioned earlier, DUBs play an important role in regulating the stability and activity of proteins, in various signal transductions, cell cycle, apoptosis, and cell proliferation among other functions [38,39,40]. The human genome encodes approximately 100 DUBs, which can be grouped into seven families, based on their sequence and domain conservation. These include: six families of cysteine proteases, namely ubiquitin-specific proteases (USPs), ovarian tumor proteases (OTUs), ubiquitin C-terminal hydrolases (UCHs), Machado–Joseph Disease protease family (MJDs), the motif interacting with ubiquitin (MIU)-containing novel DUB family (MINDYs), zinc finger with UFM1-specific peptidase domain protein/C6orf113/ZUP1 (ZUFSP), and the JAB1/MPN/MOV34 metalloenzyme family (JAMMs, also known as MPN+) which belongs to the zinc-metalloprotease group [41,42,43,44,45]. The USPs, the largest mammalian family of DUBs, have a core catalytic domain and substrate recognition domain [37,46]. Moreover, USPs have been compared to the hand with three subdomains, containing the thumb, palm, and fingers. The finger subdomain interacts with Ub to guide its positioning in the catalytic center that forms between the thumb and palm subdomains containing the catalytic Cys and His, respectively. OTUs form the second largest mammalian family of DUBs which have homology to the ovarian tumor gene (*OTU*) of fruit flies. In human, 16 members of OTUs have been identified that mainly regulate cell-signaling cascades. Generally, the OTUs are composed of catalytic OTU domain which possesses putative catalytic cysteine and histidine residues and the ubiquitin interaction domain, such as a UBA (ubiquitin associated) domain, UIM (ubiquitin interacting motif) domain, or ZnF (Zinc finger) domain [47,48,49]. The UCHs adopts a core fold and a catalytic triad resembling papain. Moreover, the UCHs possess a confined loop that cleaves short ubiquitinated peptides from the c-terminal, suggesting their important role in the recycling of free ubiquitin molecules [50,51]. The MJDs consist of four members, namely, Ataxin-3, Ataxin-3L, JOSD1, and JOSD2. All MJD family have a highly conserved cysteine protease domain, called the Josephine domain [52,53,54,55,56]. Interestingly, a recent study reported that the MJDs have a striking ubiquitin esterase activity with high specificity and significant preference to ubiquitin-threonine substrates [57]. While the preceding DUBs were reported to be less specific to Ub linkage type, JAMM metalloproteases are reported to be K63-linkage specific. For instance, AMSH, a member of the JAMM family of DUBs, is only active against K63-linked chains, primarily due to interactions with the proximal ubiquitin moiety [58,59]. Moreover, ZUFSP family member selectively interacts using tandem ubiquitin-binding domains and cleaves long K63-linked ubiquitin chains [60]. Unlike them, the recently identified MINDY family of DUBs is extremely conserved in its catalytic domain that adopts a distinct fold from any other DUBs. Notably, MINDY family prefers to cleave long K48-linked polyubiquitin chain and cleaves the distal ubiquitin component [41,61,62].

Since DUBs are proteases, regulations of deubiquitination nearly depend on expression and localization of substrate cleavages. The expression levels of DUBs in the cell can be modulated at the transcriptional level in a stimulation-dependent manner [63]. For instance, the expression level of USP29, which interacts and stabilizes cGAS, is generally low, but is upregulated upon virus infection owing to establish an antiviral state [64,65]. As mentioned above, DUB regulation is equally exerted through subcellular localization, usually necessary for and dependent on substrate accessibility [66,67]. For example, USP10, a cytoplasmic DUB, translocates to the nucleus upon DNA damage where it contributes to the stabilization of p53 [68]. Moreover, catalytic activity of DUBs can be regulated by allosteric regulation. When USP7 interacts with Ub, its HUBL-45 (HAUSP ubiquitin-like domain-45) domain allosterically binds to the switching loop in its catalytic domain. This allosteric interaction leads to full activation of the USP7 catalytic domain, thereby altering USP7 from inactive to active state [69].

## 3. RLR Signaling Modulation

RIG-I like receptor (RLR) is a family of DExD/H box RNA helicases that functions as viral RNA sensors. The RLR family includes three members: RIG-I, melanoma differentiation-associated protein 5 (MDA5), and LGP2 (laboratory of genetics and physiology 2). All RLRs contain a C-terminal domain (CTD) and an intermediate RNA helicase domain. Except for LPG2, RIG-I and MDA5 harbor the N-terminal caspase activation recruitment domains (CARDs), and the CTD which is responsible for recognition and binding of RNA [70]. Interestingly, RIG-I can detect 5′ triphosphorylated RNA as well as short double strand RNA (dsRNA), while MDA5 recognizes long dsRNA [71,72,73]. After sensing the cytosolic viral RNA, RIG-I and MDA5 activate the mitochondrial antiviral signaling protein (MAVS) that recruits several adaptor molecules including TRAF6 and TANK [74,75,76,77,78]. Subsequently, the activation and association of TBK1 and IKK-ε are triggered followed by the activation of IRF3 and IRF7, which then promotes the expression of type I IFN.

Mounting results report that many E3 ubiquitin ligases have been involved in RLR signaling at various steps of signal transduction clearly pointing out their impact on signaling [79]. For instance, TRIM25, the first identified E3 ubiquitin ligase in RLR signaling, induces the K63-linked polyubiquitination of the second CARD domain of RIG-I, leading to the conformational change enabling it to recognize cytosolic viral RNA [80]. Moreover, the E3 ligases TRIM25 and Riplet/RING finger protein 135 (RNF135) have been reported to conjugate K63-linked polyubiquitin chains to RIG-I CARD domains, thus stabilizing the RIG-I complex and boosting its activation [81,82]. TRIM65 was shown to specifically interact with and enhance K63-linked polyubiquitination of MDA5, leading to MDA5 oligomerization and activation which are essential steps for activation of IFN response. TRIM31 also causes MAVS aggregation via K63-linked polyubiquitination, leading to the activation of MAVS after viral infection [83]. Furthermore, TRIM21 interacts and induces K27-linked polyubiquitination of MAVS to promote the recruitment of TBK1 to MAVS, thereby positively regulating antiviral innate immunity [84,85,86]. Conversely to the above upregulators of the RLR-induced signaling, the RING-type E3 ligases RNF125 and RNF122 interact with RIG-I CARD domains, inducing K48-linked ubiquitination that results in proteasomal degradation of RIG-I thus dampening RLR-induced signaling [87,88]. TRIM40 and TRIM29 also facilitate the degradation of MDA5 and MAVS, by catalyzing K48-linked and K11-linked ubiquitination respectively, thus attenuating antiviral immune response [87,88,89,90]. The HECT-type E3 ligase Itch binds to MAVS via poly(C)-binding protein (PCBP) 1/2 at the C-terminal and TAX1 binding protein (TAX1BP1) at the CARD domain, which triggers K48-linked ubiquitination of MAVS and its subsequent degradation [91,92,93]. During the virus infection, MARCH5 interacts with activated MAVS oligomer and decreases MAVS-mediated IFN signaling by K48-linked ubiquitination and degradation of MAVS [94,95,96,97]. A growing body of evidence suggests that multiple E3 ubiquitin ligases tightly control RLR-induced signaling acting as either positive factors or negative factors. 

As shown in E3 ubiquitin ligases, DUBs have also been demonstrated as crucial factors in the regulation of RLR-mediated signaling. In the cases of upregulation of RLR-signaling by DUBs, deubiquitination of both RIG-I and MDA5 mediated by USP17 facilitates the induction of type I IFN [98]. USP4 also enhances RIG-I protein level and subsequently activates immune response which ultimately attenuated viral infection in Huh7.5 cells [99]. Upon HSV-1 infection, USP25 deficient mice were found to be more susceptible than wild-type (WT), because of USP25-TRAF3/TRAF6 interaction. Indeed, virus infection causes interaction between USP25 and TRAF3 (or TRAF6), leading to the stabilization of TRAF3 (or TRAF6) and thus allowing for the production of type I IFNs as well as proinflammatory cytokines [100]. Linear ubiquitin assembly complex (LUBAC), harboring two RBR-containing E3 ligases, HOIP and HOIL-1L, binds and downregulates TRIM25 at protein level as well as competes with TRIM25 for RIG-1 binding, resulting in suppression of the K63-linked ubiquitination and signaling activity of RIG-1 [101]. In contrast to LUBAC, USP15 deubiquitinates and stabilizes TRIM25, thereby upregulating RIG-1-mediated RLR signaling [102]. The K63-linked polyubiquitination of RIG-I-CARD or CTD by TRIM25 and RNF135 respectively, which is critical for activation of RLR signaling, is negatively regulated by USP21 [103]. In this light, Fan et al. showed that USP21-deficient mice and chimeric mice with USP21-deficient hematopoietic cells were more resistant to VSV infection with enhanced IFNs production compared to wild type mice [103]. CYLD inhibits RIG-I-mediated IRF3 activation and IFN production. In addition, CYLD removes polyubiquitin chains from TBK1 as well as RIG-I, suggesting CYLD is a bona fide negative factor of RIG-I associated innate immune response against viral infection [64]. Similar with CYLD, Cui J. et al. revealed that USP3 binds to the CARD domain of RIG-I and MDA5, then cleaves K63-linked polyubiquitin chains via cooperation of its zinc-finger and catalytic domains, thus negatively regulating RLR-mediated innate antiviral response [104]. After viral infection, OTUD2 (also known as YOD1) interacts with MAVS through its UBX and Znf domains at the mitochondria, followed by the cleavage the K63-linked ubiquitin chains of MAVS that induced the abrogation of MAVS oligomerization to attenuate the IFN production [105]. Moreover, OTUD1 stabilizes the E3 ligase Smurf1, allowing the degradation of MAVS, TRAF3, and TRAF6 which are crucial components of innate immune response [106]. Using DUBs library screening, OTUD4 has been identified as a positive regulator of MAVS-mediated immune response [107].

**Figure 1:**
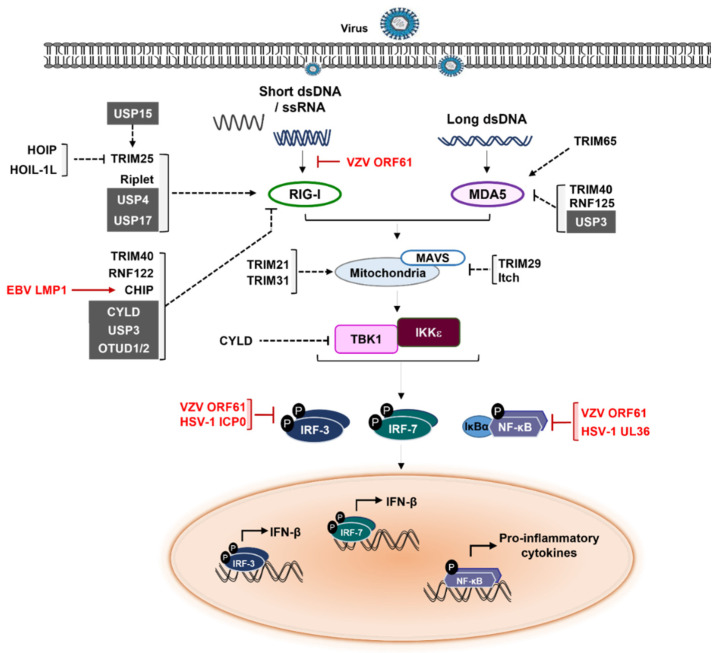
Immune evasion strategies of herpesviruses regarding E3s and DUBs in RLR-mediated signaling cascades. RIG-I recognizes and binds to ssRNA bearing a 5′-triphosphate moiety, as well as short dsRNA, leading to a conformational change that exposes CARDs. RIG-I then binds MAVS through CARD–CARD interaction. After MAVS oligomerization, it then activates IRF3/7- and NF-κB-mediated signaling by recruiting TRAFs, leading to the production of type I IFN and pro-inflammatory cytokines. The words in red color indicate herpesviruses proteins. The gray boxes represent cellular DUBs. Solid lines represent RLR-mediated signaling pathway. The dashed lines indicate effects of cellular E3 ubiquitin ligases (or DUBs) on molecules in RLR-mediated signaling pathway.

Hence, it is not surprising that herpesviruses evolutionally employ numerous strategies to subvert RLR-induced immune response. HSV-1 ICP0, a viral E3 ubiquitin ligase, inhibits host anti-viral response by blocking IRF3 (or IRF7)-mediated activation of interferon stimulating genes (ISG) [108]. HSV-1 ICP0 protein has been reported to trigger the translocation of USP7 from the nucleus to the cytoplasm, where it causes the deubiquitination of TRAF6 to terminate the TLR-mediated NF-κB activation [109]. ICP0 also dampens TNF-α-mediated NF-κB activation and inhibits TLR2-mediated NF-κB activation by inducing the ubiquitination of p50 and MyD88 respectively, thus preventing the production of NF-κB-regulated pro-inflammatory cytokines [110,111]. Collectively, these results indicate that HSV-1 has employed ICP0 to successfully establish their life cycle through manipulation of host immune response [24,111,112]. Like HSV-1, KSHV also encodes two viral E3 ligases K3 and K5 which are highly related to membrane-associated RING-CH-containing (MARCH) E3 ubiquitin ligases family [113]. Both K3 and K5 suppress immune synapse components including MHC-1 and ICAM-1 [114]. Ectopic expression of KSHV K3 (or K5) downregulates cell surface MHC I molecules via facilitated endocytosis and lysosomal degradation, but does not interfere with MHC I synthesis [115,116,117,118]. Notably, the characterization of recombinant KSHV K3 and K5 deletion mutants proved the crucial role of K5 regarding modulation of surface receptor, while K3 did not alter the surface expression of MHC-I in cells infected with K3-defective viruses, suggesting that K5, but not k3, has the major role during the infection [119]. Brulois et al. further revealed that K5 acts immediately after viral infection, while both K5 and K3 play an essential role in downregulation of surface receptor in late stage of viral infection suggesting different roles of K5 and K3 in KSHV-mediated downregulation of MHC-I molecules [114]. Interestingly, murine gamma herpesvirus 68 (MHV68) K3 and K5 also decreased the surface expression of MHC-I molecules via proteasomal degradation [113]. However, unlike KSHV, MHV68 K3 largely contributed to the downregulation of MHC-I surface expression during lytic infection [120]. The EBV viral protein LMP1 interacts with CHIP, an E3 ubiquitin ligase, and induces RIG-I degradation [121]. The varicella-zoster virus (VZV) immediate early protein ORF61 is composed of RING-finger E3 ligase domain in its N-terminus that targets phosphorylated IRF3 for proteasome-mediated degradation, thereby antagonizing the IFN-β pathway [122]. Similarly, herpesviruses have equally pirated the deubiquitination system of their host to evade RLR signaling (Figure 1). The UL36 (also called UL36USP), which corresponds to the USP family is the largest tegument protein of HSV-1. UL36 is considered as a viral DUB due to the novel DUB motif on its N-terminus. UL36 deubiquitinates TRAF3 to prevent recruitment of TBK1 and eventually downregulates or counteracts the IFN pathway [123]. When Vero cells were infected with either UL36 (C40A) mutant HSV-1 or WT HSV-1, production of IFN-β was much higher in UL36 (C40A) mutant HSV-1 than in WT HSV-1-infected cells. Intriguingly, some herpesviruses harbor homologue genes that function as DUBs; HCMV UL48, MHV68 ORF64, and KSHV ORF64 [124,125,126,127]. Even though these DUBs are highly conserved across the herpesviruses family, their roles in hijacking the host immune systems need further studies [123,128].

## 4. cGAS-STING Signaling Modulation

cGAS is a major sensor of cytosolic DNA that is also critical for launching host immune response [24]. Longer dsDNA (>20 bp) activates cGAS through promoting cGAS dimerization, allowing for rearrangement of cGAS catalytic pocket for subsequent binding of adenosine triphosphate (ATP) and guanosine triphosphate (GTP) to synthesize 2′3′-cGAMP. Subsequently, cGAMP binds and activates STING which translocates to the Golgi and stimulates STING-mediated TBK1 activation that triggers phosphorylation of IRF3 [129,130,131]. Phosphorylated IRF3 induces the transcription of IFNs as well as other inflammatory cytokines [113,116,132]. Unlike cGAS, mounting data consider IFI16 as a nuclear DNA sensor although it shuttles between the cytoplasm and the nucleus [41,133,134]. IFI16 contains a pyrin domain that activates inflammasome and two DNA-binding HIN domains that detect viral dsDNA [10,135]. IFI16 also promotes STING phosphorylation and translocation away from ER upon DNA stimulation, resulting in elevated activation of STING [136]. In addition, IFI16 was also reported to stabilize cGAMP which is necessary for STING activation. Moreover, coimmunoprecipitation assays revealed a weak interaction between IFI16 and STING. However, the mechanism used to activate STING after interaction is still not clear [136]. In HSV-1 infection, cGAS interacts and stabilizes IFI16 via conformational change that prevents its proteasomal degradation. Stabilized IFI16 then interacts with viral genome via its HIN200 domain, leading to IFN-β production [137].

Ubiquitination has also been shown to be equally important for cGAS-STING signaling. For instance, the interaction between E3 ligase RNF185 and cGAS leads to the K27-linked polyubiquitination of cGAS on Lys173 and Lys384, which promotes its enzymatic activity and subsequently enhances IRF3 expression [138]. TRIM56 induces monoubiquitination of cGAS at Lys335, thereby promoting its dimerization, DNA-binding activity, and cGAMP production that finally enhance cGAS-mediated signaling activity [139]. Like TRIM56, TRIM41 positively regulates cGAS activation by mediating its monoubiquitination [140]. Overall, monoubiquitination of cGAS has a pivotal role in the regulation of cGAS-mediated immune response by inducing IFN-α and IFN-β, but further insight is needed for better understanding. TRIM32 catalyzes K63-linked ubiquitination at Lys20, Lys150, Lys224, or Lys236 of STING, thereby activating STING that facilitates STING-TBK1 interaction [141]. Moreover, the ER-E3 ligase autocrine motility factor receptor (AMFR) in complex with insulin-induced gene 1 (INSIG1) induces K27-linked polyubiquitination of STING, which serves as an anchoring platform for recruiting TBK1 and promoting its translocation to the perinuclear microsomes thus favoring one-set of innate immune response [142]. By carrying out a limited siRNA screening, the mitochondrial E3 ubiquitin ligase 1 (MUL1) was shown to induce K63-linked polyubiquitination of STING at K224, thus initiating cytosolic DNA-mediated STING trafficking to activate the production of IFN-β and cytokines [143]. Given the distinct role of cGAS in sensing cytosolic DNA that activates the immune system, negative regulation of cGAS-STING is necessary to maintain homeostasis. This modulation has been affected by the E3 ligases RNF5, TRIM29, and TRIM30 via K48-linked ubiquitination on STING with subsequent proteasomal degradation [144,145]. RNF5 is generally localized in ER, but, after viral infection, it is translocated into the mitochondria that provides a chance to cause the STING degradation via ubiquitination at Lys150 [144]. RNF26 competes with RNF5 at Lys150, resulting in K11-linked polyubiquitination of STING to avoid STING degradation [146]. In addition, Qin et al. reported that activation of RNF26-mediated type I IFN regulation is temporal by two independent mechanisms; RNF26 interacts with STING to stabilize it during the early phase of viral infection, while RNF26 later binds to IRF3 for downregulation of IFN-mediated signaling by autophagy-associated degradation. These results suggest RNF26 might be one of the important regulatory factors used to fine-tune the host immune response [146].

USP29 has been reported to contribute to cGAS-mediated antiviral response via stabilization of cGAS through interaction between their UCH domain and the nucleotidyltransferase (NTase) upon HSV-1 infection [65]. USP20 was shown to interact with STING by removing the K48-linked ubiquitin chains from STING, owing to promote cellular antiviral responses upon HSV-1 infection [147]. Using a DUBs library, USP18 and USP49 were identified as STING-interacting proteins. Furthermore, the survival rate of WT mice infected with HSV-1 was higher than that of USP18-/- mice, suggesting USP18 is one of the crucial factors in IFN signaling [148]. More interestingly, USP18 recruits USP20 to stabilize STING via deconjugation of K48-linked ubiquitin chains from STING, thus enhancing the production of type I IFN [148]. Both USP44 and CYLD also stabilize STING through removal of K48-linked polyubiquitin chains after DNA virus infection [64,149]. USP49 interacts with and removes K63-linked ubiquitin chains from STING, leading to suppressed antiviral responses [150]. USP49-/- mice were shown to exhibit attenuated viral replication after HSV-1 infection [150]. DUBs can also cleave atypical polyubiquitin chains on STING, as demonstrated by USP13 deconjugating the K27/33-linked polyubiquitin chains from STING, which impairs the recruitment of TBK1 to STING [151]. In addition, USP22 negatively regulates STING-mediated antiviral response by interacting with USP13 to cleave the K27-linked ubiquitination of STING [152].

**Figure 2:**
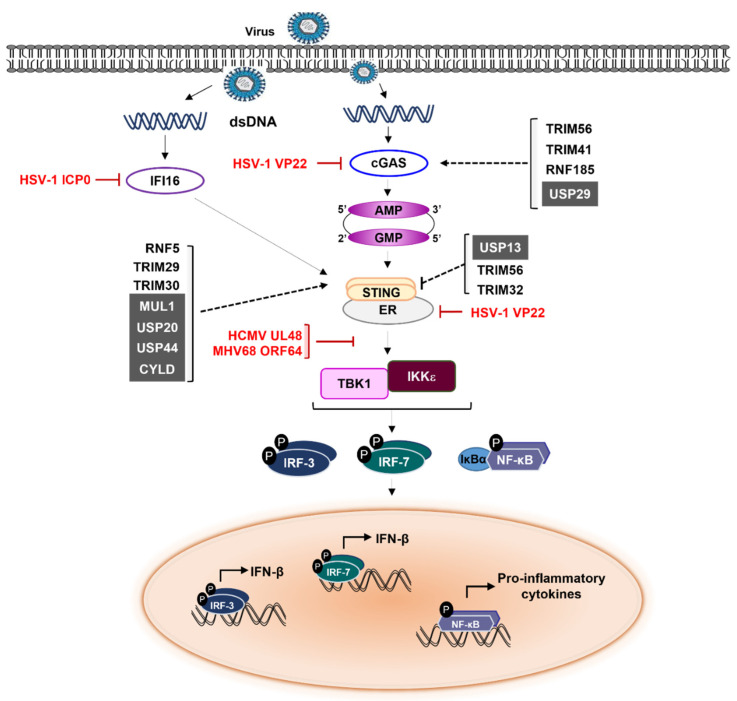
Herpesviruses escape tactics in cGAS-STING mediated signaling cascades. cGAS recognizes DNA in the cytosol and then catalyzes the formation of cGAMP. Subsequently, cGAMP binds to and activates STING. STING translocates from the ER to the Golgi, where it interacts with and phosphorylates TBK1 and IKK. IRF3/7 and NF-κB are activated by TBK1 and IKK, respectively, leading to the production of type I IFN and pro-inflammatory cytokines. The words in red color indicate herpesviruses proteins. The gray boxes represent cellular DUBs. Solid lines represent cGAS-STING mediated signaling pathway. The dashed lines indicate effects of cellular E3 ubiquitin ligases (or DUBs) on molecules in cGAS-STING mediated signaling pathway.

Viral infection-driven cGAS-STING mediated type I IFN signaling has clearly shown the crucial role of cGAS-mediated STING activation against several DNA viruses. Hence, it is notable to further confirm how DNA viruses evade cGAS-STING-mediated type I IFN signaling. EBV utilizes TRIM29 to induce K48-linked ubiquitination of STING at Lys379 site for protein degradation, thereby preventing the activation of cGAS-STING signaling and subsequently dampening the antiviral immune response [89]. Notably, TRIM29-/- mice infected with HSV-1 displayed considerably decreased viral load owing to enhanced IFN and cytokine production [89]. On the other hand, HCMV employs tegument protein UL83 which blocks DNA sensing of IFI16 by interacting with its pyrin domain to prevent the formation of a nuclear oligomer, needed to activate the IFI16-mediated STING-TBK1-IRF3 pathway [153]. HSV-1 ICP0 degrades IFI16 via its E3 ligase activity [154,155]. HSV-1 tegument protein VP22 directly binds and inhibits cGAS enzymatic activity, leading to downregulation of IFN-β production. Furthermore, effective knockdown of cGAS dramatically increased replication of ΔVP22 HSV-1 but not WT HSV-1, indicating VP22 acts as a counterpart to the cGAS-STING-mediated DNA sensing immune signaling pathway [156]. Interestingly, knockdown of IFI16 using shRNA in KSHV-positive PEL cells increased KSHV lytic reactivation, followed by viral genome replication. Correlated with this, IFI16 is degraded by ubiquitin-proteasome pathway during lytic replication, while IFI16 acts as a transcriptional repressor that suppresses lytic viral genes to maintain the latency [155]. HCMV UL48, known as viral DUBs, inhibits the type I IFN synthesis via deubiquitinating several key molecules (including TRAF6, IRF7, and STING) in anti-viral innate immunity [157]. Another viral DUBs, MHV68 ORF64 also caused the deubiquitination of STING, hence impeding the recruitment of TBK1 and downstream signaling events of the STING signalosome [158] (Figure 2). Taken together, herpesviruses have evolved several viral DUBs to evade immune response by the cytosolic DNA-sensing pathway, leading to successful establishment of latent infections.

## 5. Inflammatory Signaling Modulation

Inflammasome, a large cytosolic multiprotein oligomer, is a part of the innate immune system which is activated by either PAMPs derived from infectious microbe or damage-associated molecular patterns (DAMPs) [159]. Both PAMPs and DAMPs bind and activate PRRs, which include NLRs. Upon activation, NLRs oligomerize and then form multiprotein inflammasome complexes to activate inflammatory caspases, followed by the maturation and secretion of IL-1β and IL-18, which promotes inflammatory cell death known as pyroptosis [160]. Until now, several inflammasome complexes have been identified such as NLR-family pyrin domain (PYD)-containing 1 and 3 (NLRP1 and NLRP3 respectively), absent in melanoma 2 (AIM2), and NLR-family CARD domain containing 4 (NLRC4) inflammasomes. NLRP3, the well-studied protein among them, was reported to interact with ACS (apoptosis-associated speck-like protein containing a CARD domain) via its PYD domain and the CARD domain of ASC in turn recruits the CARD domain of pro-caspase-1 to generate NLRP3-ACS-pro-caspase-1 complex (also called NLRP3 inflammasome). This generated complex causes caspase-1 activation and IL-1β production [161]. AIM2 also recruits pro-caspase-1 via ASC to form AIM2-ASC-pro-caspase-1 complex. Unlike NLRP3 and AIM2, NLRP1 directly interacts with pro-caspase-1 in an ASC independent manner, although the presence of ASC facilitates NLRP-1 mediated caspase-1 activation [162]. Consistent with the above discussed signaling, inflammasome signaling is also regulated by E3 ligases. 

Mass spectrometry analysis and knockdown of MARCH using siRNA revealed MARCH7 to be responsible for dopamine-induced NLRP3 ubiquitination and degradation, leading to NLRP3 inflammasome inhibition [163]. The E3 ubiquitin ligase TRIM31 directly binds and ubiquitinates NLRP3 in both resting and activation conditions, thereby acting as physiological repressors of NLRP3-associated inflammasome activity [164]. The E3 ligases RNF125 and Cbl-b are essential for targeting NLRP3 for K63- and K48-linked ubiquitination, respectively, leading to NLRP3 proteasome-mediated degradation. In contrast to other K63-linked ubiquitination of NLRP3 that leads to degradation, Cullin1, the key component of the Skp1-Cullin1-F-box E3 ligase, promotes the K63-linked ubiquitination of NLRP3 preventing its activation without causing degradation [165]. Kawashima et al. demonstrated that the E3 ligase Ariadne homolog 2 (ARIH2) interacts with NACHT domain of NLRP3 and ubiquitinates NLRP3, thus negatively regulating activation of NLRP3 inflammasome in macrophages [166]. On the contrary, Pellino2, the E3 ubiquitin ligase, facilitates the maturation of NLRP3-induced inflammasome and release of IL-1β and IL-18 in macrophages [167]. 

USP7 and USP47 have been reported to have functional redundancy in deubiquitinating NLRP3. Using CRISPR/Cas9 in the THP-1 cells, inflammasome activation was reduced when both USP7 and USP47 were knocked out [168]. Concurrently, chemical inhibition of USP7 and USP47 also blocked inflammasome formation independently of transcription, by preventing ASC oligomerization and formation of ASC speck [168,169]. These results suggest USP47 and USP7 play a role as a positive regulator in NLRP3 inflammasome activation. The STAM-binding protein (STAMBP) is a member of the Jab1/MPN metalloenzyme (JAMM) family of DUBs and identified as a central regulator of the NLRP3 inflammasome through deubiquitinating NLRP3 [58]. Remarkably, STAMBP-mediated deubiquitination of NLRP3 causes inhibition of NLRP3-mediated inflammasome without stabilization of NLRP3 protein abundance [170]. The JAB1/MPN/Mov34 (JAMM) domain-containing Zn2+ metalloprotease BRCC3, a member of the BRCC36 isopeptidase complex (BRISC), directly interacts with NLRP3 and removes its K63-linked polyubiquitin chains, allowing for NLRP3 inflammasome formation [171]. ABRO1, another component of BRISC, was revealed to promote the formation of an active inflammasome complex via its deubiquitinating activity on NLRP3. This was congruent with the impaired NLRP3 inflammasome responses observed in ABRO1-/- mice [172]. In addition, ABRO1 was reported to act as a scaffold for the interaction of NLRP3 with BRCC3 and demonstrated synergic effect with BRCC3 in promoting NLRP3 inflammasome activation via regulation of NLRP3 deubiquitination [172]. A20 (also known as TNFAIP3), is the only DUB known to negatively regulate inflammasome to date and is implicated in several aspects of NLRP3 inflammasome regulation [173]. Doug et al. showed that A20-deficient macrophage promotes spontaneous NLRP3-mediated inflammasome activity in a RIPK3 (Receptor Interacting Serine/Threonine Kinase 3)-dependent manner [174].

**Figure 3:**
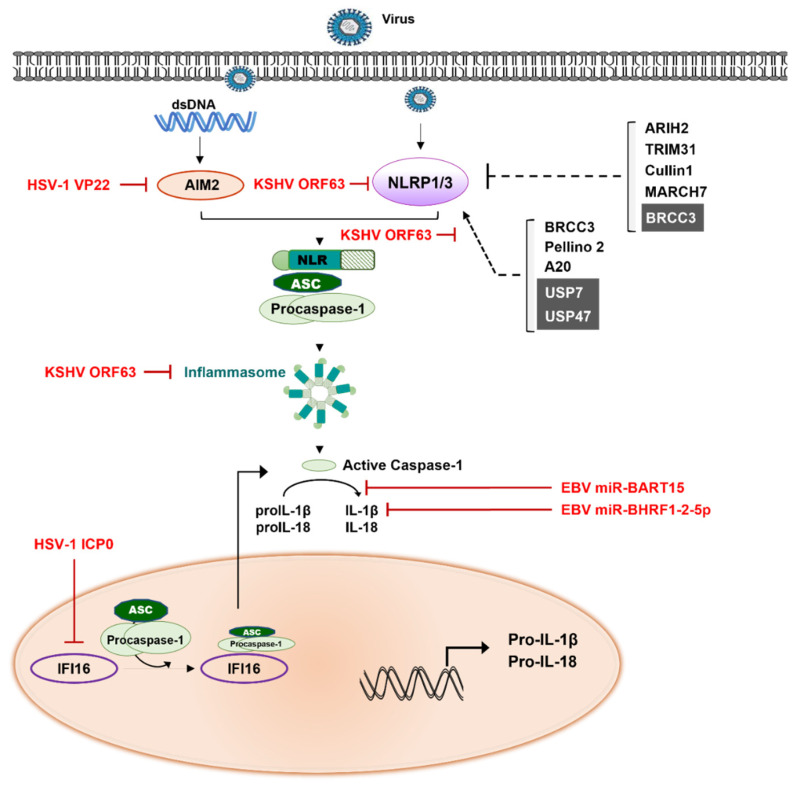
Herpesviruses-associated escape modes in Inflammasome mediated signaling cascades. The NLRP3 interacts with apoptosis-associated speck-like protein containing a CARD (ASC) via its pyrin domain (PYD) and the CARD of ASC recruits the CARD of pro-caspase-1 to generate NLRP3-ACS-pro-caspase-1 complex (also called NLRP3 inflammasome). The words of red color indicate herpesviruses proteins. The gray boxes represent cellular DUBs. Solid lines represent inflammatory signaling pathway. The dashed lines indicate effects of cellular E3 ubiquitin ligases (or DUBs) on molecules in inflammatory signaling pathway.

Hence, to perturb inflammasome signaling, KSHV ORF63 which has sequence homology with cellular NLRP1, interacts with NLRP1 and inhibits the association of NLRP1 with pro-caspase-1 to prevent the NLRP1 oligomerization [175]. Furthermore, KSHV ORF63 was found to interact with two additional NLR family members, NOD2 and NLRP3 owing to modulating NLR-mediated innate immunity for their life persistence [10,175,176]. The mechanism by which EBV mobilizes these inflammasome activators and inhibitors to its benefits is not known yet. Recently, Skinner et al. showed that EBV-encoded miR-BART15 targets the NLRP3 3′-UTR for inhibition of both NLRP3 and IL-1β maturation. In addition, EBV-infected B cells were enabled to reduce inflammasome activation via secretion of miRNA through exosomes [177]. IL-1β receptor 1 was also targeted by EBV-encoded miR-BHRF1-2-5p, thus preventing the stimulation of IL-1α and IL-1β [178]. HSV-1 tegument protein VP22 inhibits AIM2-associated inflammasome activation by preventing its oligomerization [179]. A mutant HSV-1 depleted VP22 (HSV-1ΔVP22) activates AIM2 and stimulates secretion of IL-1β and IL-18, resulting in diminished viral load in vivo, but its replication was largely restored in AIM2-deficient mice. These findings clearly show that VP22-dependent inhibition of the AIM2-mediated inflammasome activation is a critical step to establish HSV-1 replication [179]. In contrast, infection with the virulent HSV-1 strain in human corneal epithelial cells induced the NLRP3, NLRP12, and IFI16 to stimulate early inflammasome, followed by activation of caspase-1, IL-1β, and IL-18 that ultimately facilitates the recruitment of macrophage and neutrophils [180]. Hence, these results indicated that communication between host inflammasome complex and HSV-1 strains (either virulent or less virulent) determines the output of inflammasome status [181]. At early times post-infection of HSV-1, IFI16 recognizes the HSV-1 genome in the nucleus, and then re-localizes into cytoplasm, causing the ASC colocalization. On the other hand, to block NLRP3/IFI16-mediated inflammasome activation, HSV-1 ICP0 leads to the proteasomal degradation of IFI16 and HSV-1 infection occurs the trapping of caspase-1 by actin clusters [182]. Taken together, HSV-1 has evolved several mechanisms to effectively regulate antiviral and pro-inflammatory cytokines regarding inflammation complexes (Figure 3). Next, additional studies would be necessary to further understand crosstalk between inflammasome and other herpesviruses together with DUBs which are involved in inflammasome-mediated innate immune response.

## 6. Concluding Remarks

Recognition of herpesviruses by the host immune response begins through activation of diverse PRRs that produce type I IFN as well as pro-inflammatory cytokines. Thus, for every identified antiviral measure, herpesviruses employ numerous strategies to evade the innate immune response and establish their life cycle. Therefore, much work has been performed and more is still needed for better appreciation of the viral evading strategies via ubiquitination and deubiquitination. Herein, we have delineated our current knowledge on how herpesviruses manipulate the ubiquitin system to escape the host immune response, while efficiently establishing their life cycle. Especially, accumulated data show rapidly increasing examples of the human herpesviruses that not only modify ubiquitinated substrates but also employ viral ubiquitin E3 ligase as well as deubiquitinase to reshape host environment for their benefit (Table 1). Notably, identifying the substrates of these viral enzymes are still considered as a continuous challenge. In addition, the interference of these viral components has paved the way to determine the functions and substrates of these emerging viral modifiers that consequently highlight the importance of E3 ligases and DUBs in signal transduction patterning to innate immunity.

## Figures and Tables

**Table 1:** Herpesviral proteins mimicking E3 ubiquitin ligase or deubiquitinase.

Virus	Viral Protein	Mimicking Function	Role in Host Cell
KSHV	K3, K5	E3 ubiquitin ligase	Downregulates surface expression of MHC-I molecules via lysosomal degradation [115,116,117,118]
	ORF64	DUB	Suppresses RIG-I-mediated IFN signaling by reducing the ubiquitination of RIG-I [126]
	ORF63	DUB	Prevents NLRP1 oligomerization via interaction with NLRP1 [175]
HSV-1	ICP0	E3 ubiquitin ligase	Dampens and Terminate TNF-α-mediated and TLR-mediated NF-kB activation. Blocks DNA sensing via IFI16 degradation [111,182]
	UL36	DUB	Deubiquitinates TRAF3 to prevent recruitment of TBK1 [123]
HCMV	UL48	DUB	Inhibits type I IFN synthesis via deubiquitination of TRAF6, IRF7, and STING [157]
VZV	ORF61	E3 ubiquitin ligase	Antagonizes the IFN-β pathway [122]
MHV68	K3, K5	E3 ubiquitin ligase	Downregulates surface expression of MHC-I molecules via proteasomal degradation [113]
	ORF64	DUB	Impedes STING signalosome signaling via deubiquitination of STING [158]

## Data Availability

The data presented in this study are available on request from the corresponding author.

## Abbreviations

UPS	Ubiquitin proteasome system
DUBs	Deubiquitinases
RIG-I	Retinoic acid-inducible gene-I
RLR	RIG-I-like receptors
GMP	Guanosine monophosphate
AMP	Adenine monophosphate
cGAS	cyclic-GMP-AMP synthase
IFN	Interferon
HSV	Herpes simplex virus
VZV	Varicella zoster virus
HCMV	Human cytomegalovirus
HHV	Human herpesvirus
EBV	Epstein-Barr virus
KSHV	Kaposi’s sarcoma-associated herpesvirus
PRRs	Pathogen recognition receptors
PAMPs	Pathogen-associated molecular patterns
TLRs	Toll-like receptors
NOD	Nucleotide-binding oligomerization domain
NLR	NOD-like receptors
Ub	Ubiquitin
E6-AP	E6-associated protein
HECT	Homologous to E6-associated protein (E6-AP) carboxyl terminus
RBR	RING-between-RING
CRLs	Cullin-RING ligases
APC/C	Anaphase-promoting complex/cyclosome
Nedd4	Neuronal precursor cell-expressed developmentally downregulated 4
HERC	HECT and RCC1-like domain
RCC1	Regulators of chromosome condensation 1
RLDs	RCC-1-like domains
USPs	Ubiquitin-specific proteases
OTUs	Ovarian tumor
UCHs	Ubiquitin C-terminal hydrolases
MJDs	Machado-Joseph disease
MIU	Motif interacting with ubiquitin
MINDYs	MIU-containing novel DUB
JAMM	JAB1/MPN/MOV34 metalloenzyme family
AMSH	Associated Molecule with the SH3-domain of STAM
UBA	Ubiquitin associated
UIM	Ubiquitin interacting motif
ZnF	Zinc finger
JOSD	Josephin domain
ZUFSP	Zinc finger with UFM1-specific peptidase domain protein
HUBL	HAUSP ubiquitin-like domain
HAUSP	Herpesvirus-associated ubiquitin-specific protease
RNA	Ribonucleic acid
MDA	Melanoma differentiation-associated
LGP	Laboratory of genetics and physiology
CARD	Caspase activation recruitment domains
dsRNA	Double strand RNA
MAVS	Mitochondrial antiviral signaling
TNF	Tumor necrotic factor
TRAF	TNF Receptor Associated Factor
TANK	TRAF Family Member Associated NF-kB Activator
TBK1	TANK-binding kinase
IKK	I-kappa B kinase
IRF	Interferon regulatory factor
TRIM	Tripartite motif
PCBP	Poly(C)-binding protein
VSV	Vesicular Stomatitis Virus
HOIP	HOIL-1L interacting protein
HOIL-1L	Heme-oxidized IRP2 ubiquitin ligase-1
LUBAC	Linear ubiquitin assembly complex
CYLD	Cylindromatosis tumor suppressor
OTUD	OTU domain
ISG	Interferon stimulating genes
CHIP	Carboxy-terminus of Hsc70-interacting protein
LMP	Latent membrane protein
MHC	Major histocompatibility complex
ORF61	Open reading frame61
IFI16	Interferon-Inducible Protein 16
ER	Endoplasmic recticulum
AMFR	Autocrine motility factor receptor
INSIG1	Insulin-induced gene 1
MUL1	Mitochondrial E3 Ubiquitin Ligase 1
NF-kB	Nuclear Factor Kappa B
PEL	Primary effusion lymphoma
DAMPs	Damage-associated molecular pattern
NLRP1	NLR-family pyrin domain (PYD)-containing 1
AIM2	Absent In Melanoma 2
ACS	Apoptosis-associated speck-like protein containing a CARD domain
Cbl	Casitas B-cell lymphoma
BRCA1	Breast cancer 1
BRCC3	BRCA1-BRCA2-containing complex
BRISC	BRCC36 isopeptidase complex
ABRO1	Abraxas brother protein 1
RIPK3	Receptor-interacting serine/threonine-protein kinase 3
